# Inhibition of the CCL5/CCR5 Axis against the Progression of Gastric Cancer

**DOI:** 10.3390/ijms19051477

**Published:** 2018-05-16

**Authors:** Donatella Aldinucci, Naike Casagrande

**Affiliations:** Department of Molecular Oncology, CRO Aviano National Cancer Institute, via F. Gallini 2, I-33081 Aviano, Italy; naike.casagrande@libero.it

**Keywords:** CCL5, CCR5, gastric cancer, tumor microenvironment, invasion, CCR5 antagonists

## Abstract

Despite the progress made in molecular and clinical research, patients with advanced-stage gastric cancer (GC) have a bad prognosis and very low survival rates. Furthermore, it is challenging to find the complex molecular mechanisms that are involved in the development of GC, its progression, and its resistance to therapy. The interactions of chemokines, also known as chemotactic cytokines, with their receptors regulate immune and inflammatory responses. However, updated research demonstrates that cancer cells subvert the normal chemokine role, transforming them into fundamental constituents of the tumor microenvironment (TME) with tumor-promoting effects. C-C chemokine ligand 5 (CCL5) is a chemotactic cytokine, and its expression and secretion are regulated in T cells. C-C chemokine receptor type 5 (CCR5) is expressed in T cells, macrophages, other leukocytes, and certain types of cancer cells. The interaction between CCL5 and CCR5 plays an active role in recruiting leukocytes into target sites. This review summarizes recent information on the role of the CCL5 chemokine and its receptor CCR5 in GC cell proliferation, metastasis formation, and in the building of an immunosuppressive TME. Moreover, it highlights the development of new therapeutic strategies to inhibit the CCL5/CCR5 axis in different ways and their possible clinical relevance in the treatment of GC.

## 1. Introduction

Several research findings suggest that unresolved pathogen infections and chronic inflammation promote tumor development. Hence, inflammation has become another hallmark of cancer [1,2,3]. Indeed, inflammatory cellular effectors and cytokines within the tumor microenvironment (TME) can promote an antitumor immune response or support tumor pathogenesis [3,4,5]. Thus, the new challenge is to find drugs or drug combinations that are capable of counteracting the pro-tumorigenic effects of the TME or its formation [6].

Tumor cells can promote the formation of an immunosuppressive/protective TME by recruiting and then “educating” monocytes, myeloid cells, or T cells to become immunosuppressive tumor-associated macrophages (M2-TAM) [7], myeloid-derived suppressor cells (MDSC), T-regulatory cells (T-reg) [8], and mesenchymal stromal cells (MSCs) [9] capable of suppressing T and natural killer cells (NK) responses [10]. Consistently, the presence of inflammatory cells and high amounts of inflammatory mediators (e.g., cytokines, chemokines, enzymes) in the primary tumor is often associated with a bad prognosis and an increased capability to form metastasis [4,11,12].

Tumor cells and the TME can communicate through direct contact and/or through paracrine signals [6], including cytokines and chemokines, which are considered to be key orchestrators not only in inflammation and immune surveillance, but also in cancer progression [13,14] since they can act as survival/growth factors [15,16], improve angiogenesis [17], affect tumor immunity, and influence therapeutic outcomes in patients [18].

A variety of chemokines and chemokine receptors has been detected in neoplastic tissues [1,6]. We will focus our attention primarily on the C-C chemokine ligand 5 (CCL5), also known as RANTES (Regulated upon Activation, Normal T cell Expressed, and Secreted), and its receptor, C-C chemokine receptor type 5 (CCR5). CCL5 belongs to the C-C chemokine family whose members also include CCL3(MIP-1α) and CCL4(MIP-1β) [19]. CCL5, a target gene of nuclear factor kappa-light-chain-enhancer of activated B cells (NF-κB) [20,21], is expressed by T lymphocytes, macrophages, platelets, synovial fibroblasts, tubular epithelium, and certain types of tumor cells [19]. 

CCL5 induces the recruitment of different leukocyte types, including T cells, monocytes/macrophages, eosinophils, and basophils to sites of injury and infection. In collaboration with IL-2 and IFN-γ which are released by T cells, CCL5 induces the activation and proliferation of particular NK cells to generate C-C chemokine-activated killer cells [19]. 

CCL5 activity is mediated mainly by binding to CCR5, but also to CCR1 and CCR3 [19]. CCR4 [20,22] and CD44 are auxiliary receptors for CCL5 [22,23]. CCR5 is a promiscuous receptor that binds with high affinity CCL5, CCL3, and CCL4. CCR5 is the major co-receptor for HIV cell entry [24], and this property has significantly boosted the research on CCR5 antagonists/inhibitors [25].

## 2. The CCL5/CCR5 Axis in Cancer: General Mechanisms

### 2.1. CCL5–CCR5 Interactions May Favor Tumor Development in Multiple Ways

#### 2.1.1. Proliferation

CCL5 can increase cancer cell growth [15,18,26,27]. It stimulates cell proliferation by inducing the mammalian target of rapamycin (mTOR) pathway followed by a rapid upregulation of cyclin D1, c-Myc, and Dad-1 expression, or by enhancing glucose uptake with increased ATP production and glycolysis [28]. CCL5 may act indirectly by recruiting the TME, monocytes/macrophages, or fibroblasts that, in turn, may promote and sustain tumor cell survival/proliferation [14,29].

#### 2.1.2. Immunosuppression

Tumor-associated macrophage (TAM)s are a heterogeneous population of myeloid cells that contribute to immunosuppression, favoring the establishment and persistence of solid tumors as well as metastatic dissemination. The immunosuppressive effect of TAMs stems from their enzymatic activities and their production of anti-inflammatory cytokines, such as indoleamine 2,3-dioxygenase (IDO), interleukin-10 (IL-10), and transforming growth factor β (TGF-β), which have inhibitory effects on tumoricidal lymphocytes and expand T-reg populations [30]. Consistently, Halama et al. found that blocking the CCR5/CCL5 axis with the CCR5 antagonist Maraviroc (MVC) in functional organoids derived from metastatic colorectal cancer (CRC) patients, determined a macrophage repolarization with anti-tumoral effects. Myeloid-derived suppressor cells (MDSCs) are a heterogeneous population of myeloid cells that can limit productive immune responses against tumors [31]. Targeting the autocrine CCL5/CCR5 axis with MVC was found to reprogram the MDSCs and reinvigorate the antitumor immunity [32].

#### 2.1.3. Angiogenesis

Angiogenesis is a prerequisite for tumor growth and invasion [33]. CCL5 exerts proangiogenic effects by promoting endothelial cell migration, spreading, neovessel formation, and vascular endothelial growth factor (VEGF) secretion. Moreover, tumor cells, upon CCL5 stimulation, can produce VEGF or, by secreting CCL5, may recruit CCR5-expressing TAMs [19,34]. In turn, by secreting VEGF, TAMs can induce angiogenesis [18,30,35]. Thus, targeting tumor-promoting TAMs, which are now considered to be the major players in the regulation of tumor angiogenesis, may represent an attractive new therapeutic strategy.

#### 2.1.4. Migration (Metastasis Formation)

The binding of chemokines to their G-protein-coupled receptors (GPCRs) activates a series of downstream effects that facilitate receptor internalization and signal transduction, leading to integrin activation (adhesion) and polarization of the actin cytoskeleton [36]. The consequences are directional sensing, cell polarization, accumulation of the small GTPases Rac and Cdc42 and of PI3K at the leading edge, actin polymerization, and F-actin formation. These changes cause actomyosin contraction, tail retraction, and, finally, cell migration [36]. More specifically, in lung cancer, CCL5 contributes to the activation of the αvβ3 integrin and to cell migration through PI3K/Akt, which in turn activates IKKalpha/beta and NF-κB [37]. In ovarian cancer, CCL5 can induce matrix metalloproteinases-9 (MMP-9) secretion by monocytes, which, by degrading the matrix, allows for tumor cell extravasation [38]. In prostate cancer, CCL5 promotes invasion by increasing the secretion of both MMP-2 and -9 and by activating extracellular signal–regulated kinases (ERK) and Rac signaling [39]. In osteosarcoma, CCL5/CCR5 interactions act via MEK, ERK, and then NF-κB, resulting in the activation of αvβ3 integrin [40].

**Figure 1:**
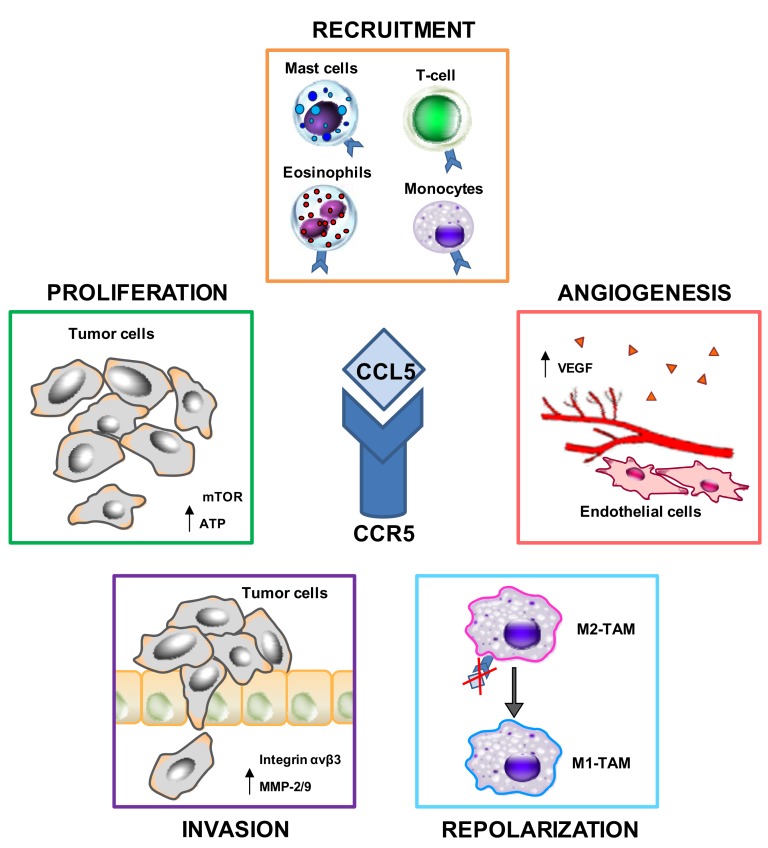
Effects of the C-C chemokine ligand 5 (CCL5) and C-C chemokine receptor type 5 (CCR5) interactions on cancer. CCL5 secreted by tumor cells or by cancer-associated fibroblasts (CAFs) recruits monocytes, T cells, eosinophils, and mast cells in the tumor microenvironment (TME). CCL5 induces tumor cell proliferation via the mammalian target of rapamycin (mTOR) pathway and increases ATP production, enhances tumor cell migration/invasion through αvβ3 integrin activation and matrix metalloproteinases-2/9 (MMP-2/9) upregulation, promotes angiogenesis by inducing vascular endothelial growth factor (VEGF) secretion; targeting the CCR5/CCL5 axis reprograms the immunosuppressive M2-tumor-associated macrophage (TAM) to anti-tumoral M1-TAM. Thin arrow, up-regulation; bold arrow, repolarization; red cross, inhibition.

A schematic representation of the consequences of the CCL5/CCR5 interactions in cancer is shown in Figure 1.

## 3. Possible Clinical Applications: CCL5 and CCR5 as Therapeutic Targets

One of the strategies in cancer therapy is to counteract the formation of a pro-tumorigenic and immunosuppressive TME. There is evidence suggesting possible clinical applications of drugs that are capable of inhibiting the CCR5/CCL5 axis or decreasing CCL5 production/secretion by tumor cells or by the TME [18].

Moreover, CCL5 levels, as well as their changes in liquid biopsy samples, could potentially be useful to monitor or predict disease progress and treatment outcomes. Clinical evidence has revealed that elevated levels of tissue or plasma CCL5 are markers of an unfavorable outcome in patients with breast [41,42,43,44], cervical [45], prostate [26], ovarian [46], gastric [47,48], colorectal [49], or pancreatic cancer [50].

### 3.1. Inhibition of CCL5–CCR5 Interactions

Finding new therapies for cancer patients is necessary, however the discovery of safe and efficacious drugs remains expensive and time-consuming [51,52]. Thus, several non-oncology drugs have been successfully repurposed for cancer [52], including the CCR5 antagonists TAK-779, Anibamine, and, especially, MVC [18,53].

MVC is a U.S. Food and Drug Administration (FDA)-approved CCR5 antagonist, which is highly selective and well tolerated, originally developed for HIV patients as a viral entry blocking inhibitor. Recently, it has demonstrated its potential to treat different types of cancer (Table 1). 

In breast cancer, MVC decreased the migration of CCR5^+^ regulatory T cells, reduced metastatic breast cancer growth in the lungs [55,67], and enhanced cell killing mediated by DNA-damaging chemotherapeutic agents [54]. In human colon cancer, it reduced the accumulation of fibroblasts in the tumor [68]. Recently, Halama et al. [53] demonstrated that T cells at the invasive margins of human CRC liver metastases produced CCL5 which had tumor-promoting effects and was responsible for the functional reprogramming/education of immunosuppressive TAMs toward a pro-tumorigenic phenotype. In a phase I trial in patients with liver metastases of advanced refractory CRC, MVC confirmed antitumoral potency [53], since treatment with the drug was associated with mitigation of tumor-promoting inflammation within the tumor tissue and objective tumor responses [53]. 

TAK-779, a quaternary ammonium derivative, is a non-peptide CCR5 antagonist with a small molecular weight, that binds exclusively to CCR5. It inhibited HIV infection and CCL5-induced proliferation and invasion of prostate cancer cells (PCa) [18].

Anibamine is the first natural product reported as a CCR5 antagonist. It produced significant inhibition of both PCa and ovarian (OVCAR-3) cancer cell line proliferation and suppressed adhesion, invasion, and tumor growth in mice [62,63].

A detailed list of inhibitors of the CCL5/CCR5 axis used in preclinical studies and clinical trials in cancer and HIV patients is shown in Table 1.

### 3.2. Inhibition of CCL5 Secretion

The inhibition of CCL5 secretion by cancer cells or by TME may represent an additional system to affect tumor progression [18]. In classical Hodgkin lymphoma, the PI3Kδ-specific inhibitors GS-1101 [17] and Auranofin [69] and the NF-κB inhibitor dehydroxymethylepoxyquinomicin (DHMEQ) [70] not only were cytotoxic, but also decreased CCL5 secretion by cancer cells, leading to a reduced capability to recruit peripheral blood mononuclear cells (PBMCs) [69].

Another therapeutic modality that deserves some consideration deals with the possibility to counteract the cross talk mediated by the CCL5/CCR5 axis between cancer cells and MSCs. 

Breast cancer cells stimulated de novo secretion of the chemokine CCL5 from mesenchymal stem cells, which then acted in a paracrine fashion on the cancer cells to enhance their motility, invasion, and metastasis [71]. Zoledronic acid (ZA) [72], as well as PEGylated nanoparticles (NPs) encapsulating ZA [73], decreased both CCL5 and IL-6 secretion by MSCs, suggesting that ZA may exert antitumor activity by affecting the ability of MSCs to interact with breast cancer cells. 

Along this line, we recently found that the epidermal growth factor receptor (EGFR) tyrosine kinase inhibitor, gefitinib decreased the capability of supernatants from PCa cells to increase CCL5 secretion by MSCs [74].

Overall, decreasing cancer cells or TME secretion of CCL5 using anticancer drugs may affect both tumor cell proliferation and/or the formation of a protective/immunosuppressive TME.

## 4. Gastric Cancer and Its TME

Gastric cancer (GC) is the fifth most common cancer worldwide [75]. The precise pathogenesis of GC remains unclear. It has been correlated to many factors, such as eating habits, environmental factors, hereditary predisposition, chronic gastritis, gastric polyps, gastric mucosa abnormal hyperplasia, and *Helicobacter pylori* (*H. pylori*) infection. At diagnosis, over 50% of patients present locally advanced or metastatic GC and consequently are ineligible for curative surgery. When surgery is not possible, chemotherapy is often given to reduce tumors, but with low benefit to patients. Therefore, to improve GC treatment, it is fundamental that we find the molecular events that are responsible for the development and progression of this malignancy [76,77].

Inflammation plays a decisive role at different stages of tumor development, including initiation, promotion, malignant conversion, invasion, and metastasis [1,2,77,78,79,80]. *H. pylori*, a microaerophilic gram-negative bacterium that colonizes the gastric mucosa of 50% of the human population, plays a predominant role in the etiology of GC [81]. Its carcinogenic potential is driven by the interplay between bacterial virulence factors and the host’s immune responses that allow *H. pylori* to switch between commensalism and pathogenicity. The result is chronic inflammation, with the production of cytokines/chemokines and cell proliferation, which increases the risk of DNA damage and, consequently, tumorigenesis [81]. According to the strong association between infections with *H. pylori* and neoplastic transformation in the human stomach, *H. pylori* has been classified as a class I carcinogen by the World Health Organisation in 1994, representing the strongest known risk factor for GC [81,82]. While many virulence factors of *H. pylori* have been described, the CagA (cytotoxin-associated gene A) toxin, which is translocated into gastric epithelial cells via a bacterial secretion system, appears to be the most specific for the development of a pathological phenotype. Infection with *H. pylori*, a potent activator of NF-κB in gastric epithelial cells, increases CCL5 [47,81,82,83] and induces the expression of a variety of genes, including IL-1, IL-6, IL-8, IL-10, TNF-α, VEGF, cyclooxygenase-2 (COX-2), inducible nitric oxide synthase (iNOS), cell cycle regulators, the matrix metalloproteinases (MMP)-2, MMP-7, MMP-9, and also adhesion molecules [82,84].

The chronic inflammatory state of the stomach, caused by *H. pylori* infection as well as the production of inflammatory mediators, cytokines, and chemokines, such as CCL5 within gastric tissues, plays an important role in the initiation and progression of GC. Furthermore, in GC, tumor cell survival, growth, proliferation, and metastasis are promoted by the interaction with the TME [84]. The TME of GC is composed of many different types of cells, including TAMs, lymphocytes, cancer-associated fibroblasts (CAFs), and endothelial cells [84].

### 4.1. Macrophages (TAMs)

Monocytes from the peripheral blood are recruited in the TME and differentiate into TAMs in response to chemokines, including CCL5, and growth factors produced by stromal and tumor cells [30]. In GC, TAMs can improve genetic instability, promote cancer stem cells [85], increase metastasis, and contribute to the formation of an immunosuppressive TME by inhibiting T cell activation [86,87]. Thus, inhibition of monocytes/macrophage recruitment and/or survival in tumors or their immunosuppressive reprogramming may also represent a new therapeutic option for GC. Indeed, TAM levels into GC tumor tissue directly correlate with tumor vascularity [84] and the strength of tumor invasion, nodal status, and clinical stage [84,87]. 

### 4.2. Regulatory T Cells (T-Regs)

T-regs are functionally immunosuppressive subsets of T cells, and play an important role in immunological self-tolerance [88]. T-reg (FOXp3^+^) cells have been identified as regulatory components of the adaptive immune response and are associated with *H. pylori*-related inflammation and bacterial persistence [89]. The frequency of T-regs among tumor infiltrating lymphocytes (TILs) derived from tumor-draining regional lymph nodes or peripheral blood lymphocytes is higher in GC than in normal gastric tissue [84,89]. Patients with a higher proportion of T-regs showed poorer survival rates than those with a lower proportion. Interestingly, after patients underwent curative resection for GC, the proportion of T-regs decreased and came back to levels comparable to those for normal, healthy donors [89]. Thus, naturally occurring Foxp3^+^ T-regs may be induced to migrate from the peripheral blood to the tumor sites by the chemokines CCL17, CCL22, and CCL5 and then increase in number by tumor-related factors to create a favorable environment for tumor growth [89].

### 4.3. Cancer-Associated Fibroblasts (CAFs)

CAFs are important components of various types of tumors, including GC [90,91]. During tumorigenesis and progression, CAFs play critical roles in tumor invasion and metastasis via a series of functions, i.e., extracellular matrix deposition, metabolism reprogramming, and chemoresistance [90,91]. CAFs may modulate several aspects of tumor biological behavior in GC, including the ability to proliferate, metastasize, and invade. Additionally, CAFs increase the infiltration of immune cells into GC stroma and increase the rate of angiogenesis by secreting VEGF [92].

### 4.4. Endothelial Cells (Angiogenesis)

Angiogenesis is the result of an imbalance between positive and negative angiogenic factors released by tumor and host cells into the TME. In GC, angiogenesis is promoted by *H. pylori* [93], high numbers of CAFs [77,92], and TAMs [94,95]. In addition, both GC tumor and stromal cells produce various angiogenic factors, including VEGF, IL-8, and platelet-derived endothelial cell growth factor (PD-ECGF). Tumor angiogenesis plays an essential role in growth, invasion, and metastatic spread of GC [96], indicating that pharmacologic blockade of angiogenesis is a promising new therapy, and that the real-time assessment of the vasculature status is a promising approach to predict the efficacy of the treatments and improve the clinical management of patients with GC [97]. Indeed, high levels of angiogenic factors in serum and tumors are associated with worse outcomes in GC patients. VEGF-A, the most extensively studied angiogenic factor, appears to be a useful biomarker for disease progression and remission, but not for diagnosis [96].

## 5. The CCL5/CCR5 Axis in GC Development and/or Progression

GC is a common gastrointestinal tumor characterized by rapid lesion development and poor prognosis. Diagnosis of GC is difficult because most patients are asymptomatic in the early stages of disease, which leads to a delay in treatment [81]. Therefore, early diagnosis of GC is essential, and cytokines detection is now regarded as a potential diagnostic tool. 

Existing literature highlights the fundamental role of CCL5 in GC progression. GC patients have significantly higher serum CCL5 levels compared with control groups [47,98]. The overall survival of patients with CCL5 levels higher than 71 pg/mL was found to be significantly lower than that of patients with less CCL5 [47,99]. Higher CCL5 levels were associated with lower histological differentiation, higher depth of tumor invasion, more frequent lymph nodes involvement, and advanced tumor stage [99]. More recently, a retrospective analysis of 105 patients with GC demonstrated that increased CCL5 serum levels correlated with more advanced T and N stages, poorly- or undifferentiated histological types, peritoneal metastasis, higher rates of residual tumor, and shorter survival [100]. 

Patients in the high CCL5 group also had stronger CCL5 immunohistochemistry (IHC) staining in tumor tissues [47,98] and in metastatic lymph nodes [101]. Thus, high CCL5 serum levels, along with strong IHC (CCL5) staining and poorly- or undifferentiated cancer, may be used to predict peritoneal dissemination and a poorer prognosis [100]. 

A novel prognostic gene expression risk score, including the expression of CCL5, CTNNB1, EXOSC3, LZTR1, and clinical parameters, was recently established and validated for perioperative chemotherapy treatment of GC [102]. CCL5 was also included among genomic markers that could be useful predictors of chemotherapy efficacy for better prognosis and survival outcomes in GC [103]. High expression of the *CCL5* and *CXCL12* genes in Lauren’s diffuse type of GC and increased expression of ADAMTS1, CXCL12, and *CCL19* genes were found in peritoneal metastasis, suggesting their involvement in tumor progression [103]. 

Human GC cell lines characterized by a high metastatic potential have increased CCL5 expression levels [104]. In vitro studies demonstrated that supernatants from highly metastatic GC cell lines increased CCL5 expression in PBMCs. In turn, GC cells cultured with PBMCs had higher invasion properties, and this process was inhibited by neutralizing anti-CCL5 antibodies [105]. 

Sugasawa et al. [106] demonstrated that CCL5 is expressed by TILs (CD4^+^ rather than CD8^+^ cells) and CCR5 is expressed by GC cells. CD4^+^ cells, but not CD8^+^ cells, cocultured with GC cells (MKN45 and KATO III cell lines) remarkably enhanced CCL5 production in a direct cell–cell contact manner [106]. Treatment of GC cells with CCL5 increased the proliferation and cocultivation of CCL5-treated GC cells, and PBMCs decreased the proportion of CD8^+^ cells but not CD4^+^ cells, suggesting a Fas-FasL-mediated apoptosis in CD8^+^ cells. In immunodeficient mice coinjected with KATO III and PBMCs, neutralization of CCL5 decreased tumor growth, suggesting that GC cells may induce CD4^+^ T cells to secrete the tumor-promoting CCL5 and may inhibit the anticancer activity of CD8^+^ cells [106].

CAFs represent the prominent stromal cellular components in the GC TME [92,107]. The Krüppel-like factor (KLF) KLF5 is a DNA-binding transcriptional regulator that is involved in the tumor-initiating properties of cancer stem-like cells, migration, and drug resistance [108]. In GC patients, high levels of KLF5 in CAFs were closely associated with clinical pathological features such as tumor size, invasion depth, cell grade, and lymph node metastasis, as well as poor prognosis [109]. Yang T et al. demonstrated that the upregulation of KLF5 in CAFs promoted tumor growth, migration, and invasion of GC cells in vitro and in vivo. The major factor contributing to these effects was the increased secretion of CCL5 due to KFL5 in CAFs. Moreover, they found that CCR5 expression in GC cells was activated by CCL5 produced by CAFs. Since the downregulation of KLF5 in CAFs inhibited GC cell progression, KLF5 and/or the CCL5/CCR5 axis may represent promising targets for the treatment of GC [109]. 

Monocytes/macrophages, which are crucial drivers of tumor progression, express the CCR5 receptor [30]. Consistently, a significant positive correlation was found between the expression of CCL5 and CD68 (macrophage marker) in GC tissues [85]. High levels of CCL5 and CD68 are associated with tumor size, degree of tumor invasion, lymphatic metastasis, pathological grading, and tumor thrombus, but are unrelated to patient age and gender [85]. 

In addition, Ding et al. also [98] found that CCL5 and CD68 expression are positively correlated, were highly expressed in GC tissues, and were associated with the depth of invasion, lymph node metastasis, TNM staging, and tumor differentiation. In vitro experiments demonstrated that the co-cultivation of GC cells with THP-1 used as a model for monocytes/macrophages, increased CCL5, MMP2, and MMP9 in THP-1 cells [98] and increased proliferation, clone-forming ability, and movement/migration in GC cells (also enhanced by exogenous CCL5) [98]. Thus, the authors suggested that, by secreting CCL5, TAMs promote GC cell proliferation, invasion, and metastasis. 

In conclusion, CCL5 may represent a marker of GC staging, disease progression, and a new therapeutic target [98].

## 6. Possible Clinical Applications of MVC in GC

The CCL5/CCR5 axis is a potential therapeutic target in different cancer types. Since several studies have demonstrated its involvement in GC progression [48,101,106,110], counteracting the pro-tumorigenic effects of the CCL5/CCR5 axis with CCR5-antagonists, such as MVC [53,111], or alternatively, with drugs that are capable to of decreasing CCL5 secretion [69] may be a new therapeutic options for GC treatment. 

By using anti-CCL5 antibodies, Cao et al. [92] reverted chemotaxis of GC cells induced by protein extracts from GC lymph nodes harboring metastasis, suggesting that CCL5 and CCR5 contribute to the migration of GC cells from primary to metastatic sites.

In another study, Mencarelli et al. demonstrated that MKN45, MKN74, and KATOIII GC cell lines at different stages of differentiation expressed both CCR5 and CCL5 and that MVC reduced tumor cell migration induced by CCL5 and adhesion to the explanted murine peritoneum [110]. MVC treatment decreased tumor xenograft growth of MKN45 GC cells and the extent of peritoneal disease and increased mice survival. Thus, the CCR5/CCR5-ligand axis seems to be involved in GC cell dissemination, suggesting anticancer potential of CCR5 antagonists [110]. 

Consistently, DT-13, a saponin of dwarf lilyturf tuber (Table 1), was found to inhibit BGC-823 and HGC-27 GC cell lines migration through downregulation of both CCR5 and CCL5 expression [64].

More recently, using CCR5 antagonists, Wang et al. demonstrated the involvement of CCL5/CCR5 signaling in the cross-talk between GC cells and TAMs leading to tumor growth [112], providing an additional link between inflammation and GC. Chronic inflammation can promote tumor progression via aberrant DNA methylation, an epigenetic modification [113] in neoplastic cells. DNA methylation is catalyzed by enzymes of the DNA methyltransferase (DNMT) family, including DNMT1, the major DNMT in adult cells, highly expressed in GC [114]. Gelsolin (GSN) is an actin-binding protein that controls actin filament assembly and disassembly. Its expression is downregulated in many cancers, including GC tissues, which suggests that it has a potential role in tumor suppression [112]. GSN staining in gastric tumors revealed high GSN expression in early-stage GC compared with advanced-stage tumors [112]. GSN decrease was mediated by DNMT1 promoter methylation and low GSN levels, associated with high DNMT1, and predicted poor survival in GC. 

Wang et al. [112] found that TAMs infiltration in GC tissues correlated with high DNMT1 expression. Consistently, co-culture experiments demonstrated that M2-like macrophages suppressed GSN expression in GC cells by upregulating DNMT1. Using anti-CCL5 neutralizing antibodies and the CCR5 antagonist MVC, Wang et al. [112] demonstrated that co-cultivation of GC cells with macrophages increased the secretion of several cytokines, but only CCL5 (secreted by M2-like macrophages) stimulated DNMT1 expression. Moreover, treatment with 5-AZA, a potent DNMT1 inhibitor, or with the CCR5-antagonist MVC slowed GC tumor xenograft growth, revealing the antitumor effects of DNMT1 suppression by the inhibition of CCR5 engagement in GC. Thus, MVC, which is capable of disrupting CCL5/CCR5 interactions, may represent a new potential therapeutic option to counteract TAM-induced tumorigenesis [112].

**Figure 2:**
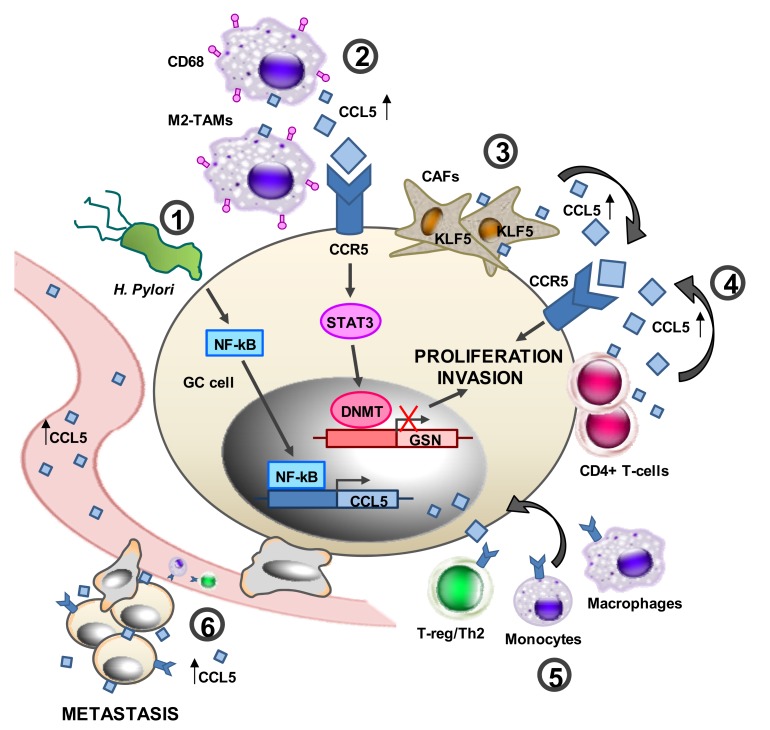
A schematic representation of the proposed role of CCL5 in gastric cancer (GC). (**1**) By activating nuclear factor kappa-light-chain-enhancer of activated B cells (NF-κB), Helicobacter pylori may induce CCL5 expression in GC cells. (**2**) By secreting CCL5, M2-TAMs may activate signal transducer and activator of transcription 3 (STAT3) and DNA methyltransferase (DNMT) and inhibit gelsolin (GSN) expression, leading to enhanced GC cancer cell proliferation and invasion/metastasis formation. CCL5 up-regulation (**3**) Krüppel-like factors 5 (KLF5) overexpression in CAFs enhances the secretion of CCL5, which induces GC cell invasion and proliferation. (**4**) By secreting CCL5, CD4+ tumor-associated lymphocytes (TILs) may enhance GC cell proliferation and invasion. (**5**) By secreting CCL5, GC cells may recruit T-regulatory cells (T-regs), monocytes, and macrophages in the TME. (**6**) Increased CCL5 in GC metastatic tissues and serum may enhance GC cell invasion. Thin up-arrow, CCL5 up-regulation; red cross, inhibition; curved arrow, binding of CCL5 to CCR5 (3, 4); curved arrow, cell migration to GC cells (5).

A schematic view of the CCL5 functions in GC and possible clinical applications of MVC are shown in Figure 2.

## 7. Conclusions

Collectively, several studies suggest that the CCL5/CCR5 axis is associated with GC progression due to increased growth and metastasis formation, though we cannot rule out a role of CCL5 also in the formation of an immunosuppressive TME [32,53]. Our current knowledge leads us to suggest the CCL5/CCR5 axis as a potential therapeutic target in GC.

## Figures and Tables

**Table 1:** CCL5/CCR5 axis inhibitors used in preclinical studies and clinical trials (cancer and HIV).

Compound	Mechanism/Molecule	Cancer-Related Studies	References
Maraviroc Selzentry, Celsentri, UK-427857 (Pfizer) Approved by US FDA in 2007 for the treatment of HIV patients.	CCR5 antagonist	Enhanced cell killing mediated by DNA-damaging chemotherapeutic agents in breast cancer.	[54]
		Reprogrammed immunosuppressive myeloid cells and reinvigorated antitumor immunity.	[32]
		Repolarized TAMs. Objective clinical responses in advanced colorectal cancer patients with liver metastases (Phase I trial).	[53]
		Decreased migration of CCR5+ regulatory T cells, reduced breast cancer growth in the lungs.	[55]
Vicriviroc SCH 417690, SCH-D(Merck)	Pyrimidine CCR5 entry inhibitor of HIV-1	Enhanced cell killing mediated by DNA-damaging chemotherapeutic agents in breast cancer.	[54]
		Inhibited invasiveness and metastatic potential in preclinical models of breast cancer.	[56]
TAK-779 (Takeda)	CCR5 antagonist, nonpeptide, quaternary ammonium derivative	Failed to protect from developing liver metastases in mice.	[57]
		Reduced T-regs infiltration and tumor growth in a pancreatic cancer mouse model.	[58]
Met-CCL5 Met-RANTES	CCR5 inhibitor, competitive chemokine receptor blocker	Decreased mammary tumor cell invasion and activation of matrix metalloproteinases induced by mesenchymal stem cell-derived CCL9 and CCL5.	[59]
		Decreased breast tumor growth, infiltrating macrophages, increased stromal development and necrosis in mice.	[60]
OTR4120 and OTR4131	GAG mimetics, inhibit CCL5 binding to GAG	Strongly inhibited CCL5-induced migration and invasion of hepatocellular carcinoma.	[61]
Anibamine	CCR5 antagonist, natural product	Inhibited the proliferation of ovarian cancer cell lines, showing reduced cytotoxicity.	[62]
		Inhibited prostate cancer cell growth, adhesion, and invasion. Reduced tumor growth in mice.	[63]
DT-13	Steroidal saponin of dwarf lilyturf tuber	Inhibited gastric cancer cell migration by downregulation of both CCR5 and CCL5 expression.	[64]
		Inhibited breast cancer cell proliferation, adhesion, and migration and lung metastasis in vivo by reducing VEGF, CCR5, HIF-1α.	[65]
Aplaviroc (GlaxoSmithKline)	CCR5 entry inhibitor	Developed for the treatment of HIV infection. Studies of Aplaviroc were discontinued because of liver toxicity.	[66]
GSK706769 (GlaxoSmithKline)	CCR5 antagonist	2008 Completed phase I trial for HIV treatment.	https://adisinsight.springer.com/drugs/800023238
INCB009471 (Incyte Corporation)	CCR5 inhibitor	Phase of Development: II (discontinued). HIV treatment.	https://aidsinfo.nih.gov/drugs/print/516/incb-9471/0/1/professional
Cenicriviroc TBR-652, TAK-652 (Takeda)	Inhibitor of CCR2 and CCR5 receptors	Completed study in a Phase IIb clinical trial for HIV treatment.	https://www.clinicaltrials.gov/ct2/show/NCT01338883
